# Ischiorectal Mass Excision Using Ultrasound-Guided, Wire-Needle Localization

**DOI:** 10.70352/scrj.cr.25-0309

**Published:** 2025-10-30

**Authors:** Michael H. Froehlich, Niharika Singh, Deborah A. Nagle

**Affiliations:** Department of Surgery, Stony Brook University Hospital, Stony Brook, NY 11794, USA

**Keywords:** ischiorectal mass, image-guided excision, wire-needle localization, fibroma

## Abstract

**INTRODUCTION:**

In this work, we describe an unusual presentation of a nuchal-type fibroma, presenting as a mass in the ischiorectal space in an adult male. Since this lesion was not palpable and was in close proximity to the anal sphincter complex, we describe a novel multidisciplinary approach using wire-needle localization with interventional radiology to allow for a directed dissection, similar to an approach used in breast partial mastectomy surgeries.

**CASE PRESENTATION:**

A 56-year-old male presented with an incidentally found 2.1 × 2.7 × 1.8 cm ischiorectal mass that abutted the anal sphincter complex. Interventional radiology performed an ultrasound-guided wire needle localization which allowed for a directed dissection down to the lesion. Pathology was consistent with nuchal-type fibroma with negative margins. The patient now undergoes surveillance with semi-annual MRIs.

**CONCLUSIONS:**

This case report describes a novel application of a hybrid surgical approach utilizing intraoperative image-guided localization for the safe excision of a rare soft tissue mass in an atypical, difficult-to-access location.

## INTRODUCTION

Wire-needle localization is a technique commonly employed in breast surgery for the localization of non-palpable breast lesions in order to guide directed dissection.^1)^ The technique entails preoperative, radiographic (typically CT or ultrasound) guided wire placement into the breast with the goal of placing the tip of the wire into the lesion or area of concern that requires excision.^1,2)^ While superficial, palpable lesions are relatively easy to locate, deeper and/or smaller lesions that are non-palpable are often difficult to localize. Thus, this technique is utilized in order to direct the dissection of the operative surgeon without causing unnecessary cosmetic and physiologic damage to the surrounding breast tissue.^1,2)^

The application of this wire-needle localization technique to aid in excision of difficult-to-localize target lesions in other parts of the body beyond the breast is somewhat limited. Thoracic surgeons have attempted preoperative CT-guided localization of pulmonary nodules for video-assisted thoracic surgery (VATS).^3–6)^ Head and neck surgeons have utilized this technique in previously operated or radiated necks to guide dissection for thyroid and parathyroid lesions, or metastatic lymph node.^7–9)^ The technique has even been utilized for muscle and nerve biopsies.^10–12)^ However, there have been no studies to date that have demonstrated utilization of this technique in colorectal surgery, specifically with regard to perirectal lesions or nuchal-type fibromas (NTFs), the pathology of the specific lesion discussed in this case.

In this paper, we present a novel application of the wire-needle for excision of non-palpable perirectal mass that had already previously undergone failed attempted local excision. Given the close proximity of this lesion to the rectum and anal sphincter complex, surgical dissection down to the lesion carried an increased risk of iatrogenic injury. Thus, we conceived a plan to proceed with an intraoperative hybrid localization approach utilizing ultrasound-guided, wire-needle localization to guide excision, in conjunction with preoperative CT imaging. This technique not only allowed for successful localization of the lesion, but for meticulous dissection along a safe plane that would minimize the risk of iatrogenic injury. We hope that this case report and review of the literature provides insight into novel approaches for difficult-to-localize lesions, specifically in, but not limited to, the perirectal region.

## CASE PRESENTATION

The patient was a 56-year-old male with obstructive sleep apnea, obesity, and a former smoker with World Trade Center exposure, who presented to a colorectal surgery clinic after incidental finding of an ischiorectal mass. The patient previously underwent routine screening colonoscopy, where 9 polyps were found and removed. Atypical inflammation was seen on colonoscopy near the cecum, prompting a CT scan of the abdomen and pelvis that revealed an incidental cystic structure in the right ischiorectal fossa. The patient was asymptomatic, with no pain at rest, while sitting, and no changes in bowel habits.

**Fig. 1:**
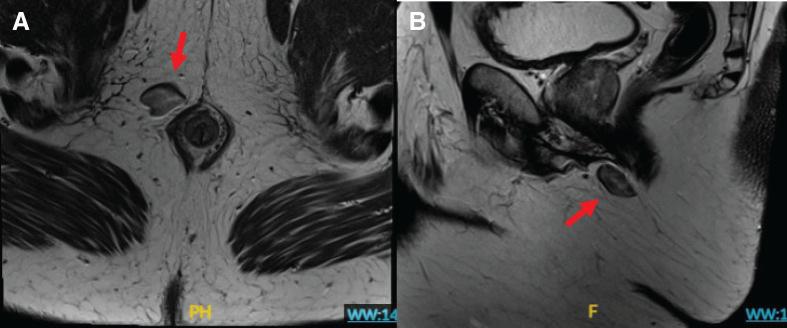
(**A**) Axial and (**B**) Sagittal slices of MRI rectum highlighting ~2.1 × 2.7 × 1.8 cm non-specific solid-cystic lesion in the right ischiorectal fossa just superior to the anal verge.

For further characterization of the lesion, MRI of the pelvis was obtained, which demonstrated a non-specific, 2.1 × 2.7 × 1.8 cm heterogeneous right ischiorectal fossa lesion with near-abutment of the sphincter complex, but without evidence of direct involvement (**Fig. 1A**, **1B**). The differential diagnoses at the time included solitary fibrous tumor, gastrointestinal stromal tumor (GIST), aggressive angiomyxoma, leiomyoma, neurogenic tumor, or sarcoma.

The patient had previously presented to an outside hospital 3 months prior, where he underwent attempted transgluteal excision; however, they were unsuccessful at localizing and excising the lesion due its deep, non-palpable nature. A specimen was obtained, however, the final pathology was read as, “adipose tissue” and repeat imaging demonstrating an unchanged lesion in the same location. Based on the appearance of the lesion that we successfully located and excised, as well as its characteristics on our final pathology, we can infer that surrounding adipose tissue and not the lesion was excised during the index case.

The patient subsequently presented to our institution for re-evaluation. Repeat MRI three months later revealed no changes in size or character of the lesion. Following multidisciplinary discussion, given the difficult-to-access location and appearance on imaging without direct extension to the rectal wall, the decision was made to forgo incisional or core needle biopsy and re-attempt transgluteal excision. However, we did not know the pathology and what, if any, acceptable margin would be required. Thus, this procedure would serve as an excisional biopsy, with plans to return to the operating room or proceed with adjuvant therapy, depending upon the final pathology.

We aimed to successfully localize and excise the mass without creating an unnecessarily large incision or damaging the anus, distal rectum, or sphincter complex. This was particularly of concern not only due to the proximity of the lesion to those structures, but also due to the deep, non-palpable nature of the lesion, making it difficult to localize. We subsequently devised a plan to have Interventional radiology (IR) perform ultrasound-guided wire-needle localization. We would then carry out dissection along the tract of the wire down to the lesion, similar to the manner in which breast masses are excised during partial mastectomy. IR was then formally consulted and a plan was finalized to perform ultrasound-guided, wire-needle localization of the mass in the operating room prior to excision.

**Fig. 2:**
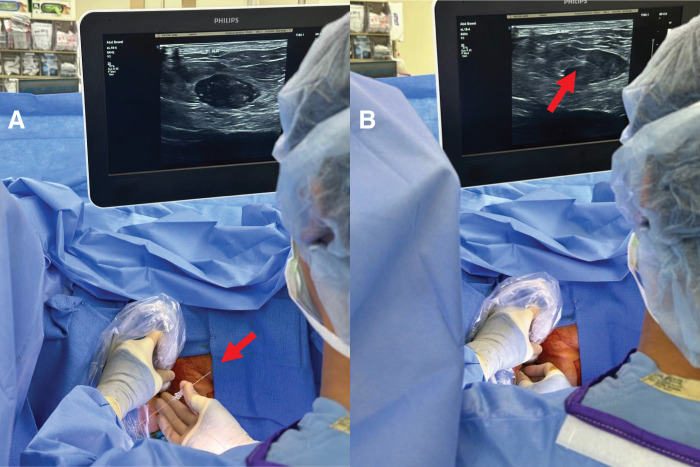
Intraoperative photograph of ultrasound-guided placement of (**A**) 15 cm, 21-gauge spring-hook wire (**B**) with tip placement within the mass to facilitate guided dissection.

The patient was subsequently taken to the operating room (OR) and placed under general anesthesia. He was then placed in lithotomy position due to the anterior position of the mass. IR then performed ultrasound-guided wire-needle localization of the mass (**Fig. 2A**). A 15 cm long, 21-gauge spring-hook wire with a needle hub was utilized. Using ultrasound guidance, the wire tip was advanced through the right anterior-medial aspect of the gluteus and advanced until the wire tip was successfully placed within the lesion (**Fig. 2B**).

**Fig. 3:**
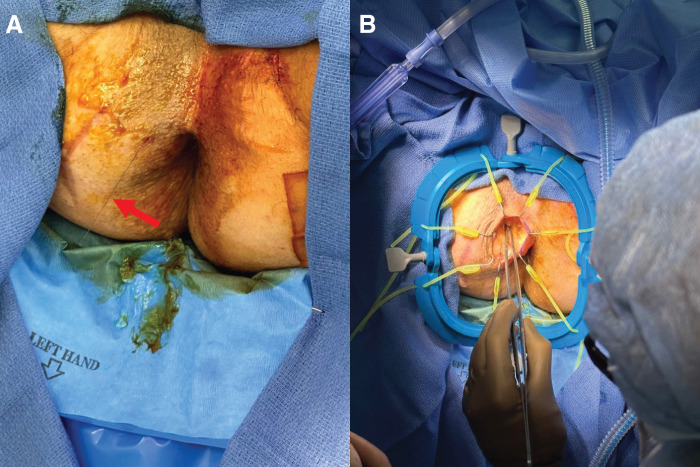
Intraoperative photograph of (**A**) wire needle in the 10 o’clock position to anus indicating trajectory of track over which (**B**) guided dissection was carried out through the subcutaneous tissue toward the lesion with the assistance of a self-retaining adjustable retractor.

**Fig. 4:**
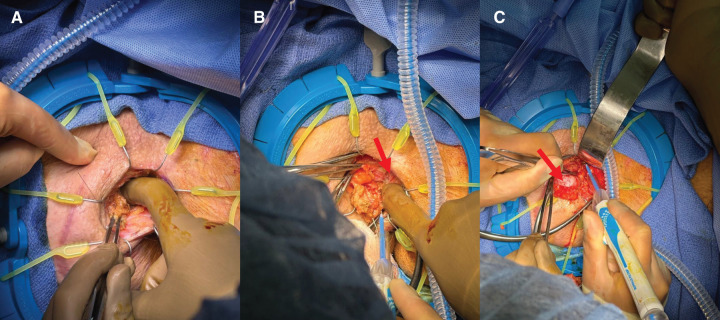
Intraoperative photograph of (**A**) dissection along wire (**B**) with frequent manual palpation to ensure dissection occurred along a safe plane between the wire and anorectal sphincter complex, with (**C**) identification of mass at tip of wire.

**Fig. 5:**
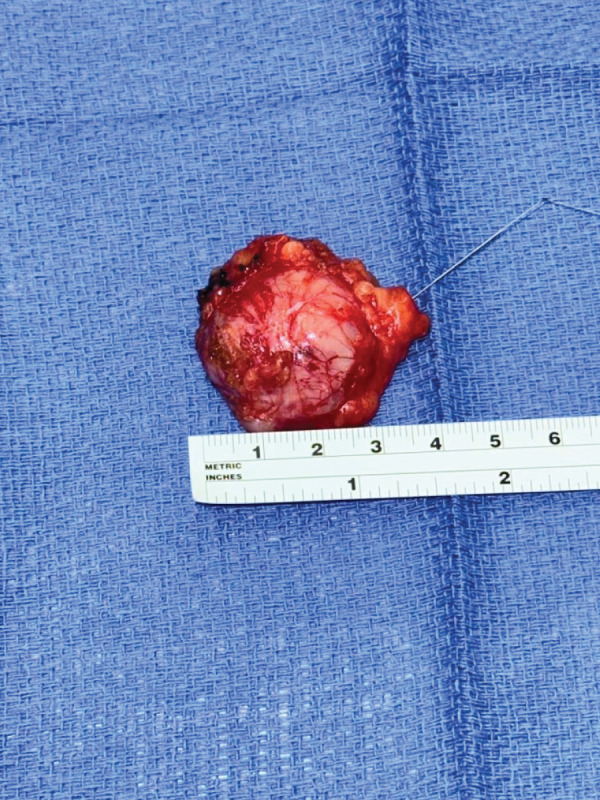
Operative photograph of excised ischiorectal mass specimen.

Bilateral pudendal block was then performed. The needle was visualized at the 10 o’clock position (**Fig. 3A**). A curvilinear incision from 9 to 12 o’clock was drawn just outside the sphincter complex. Using a self-retaining, adjustable retractor, the subcutaneous tissue was divided and the track along the needle was followed (**Fig. 3B**). As dissection was carried out, we ensured that we followed a plane between the wire and the sphincter complex to avoid iatrogenic injury (**Fig. 4A**, **4B**). Upon further dissection, the tip of the guidewire was seen just at the inferior edge of the mass (**Fig. 4C**). It was distinct in appearance from the surrounding normal gluteal fat, consistent with the radiographic findings. We carefully grasped and dissected the free neoplasm from the surrounding tissues. The lesion was successfully excised in one piece without rupture. The mass was then sent to pathology with the wire attached (**Fig. 5**). Hemostasis was ensured in the field and the wound was closed in multiple layers.

**Fig. 6:**
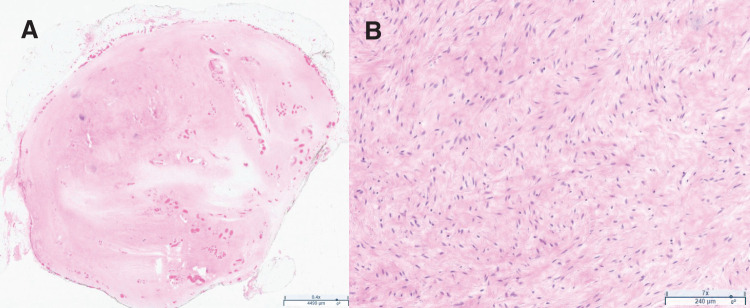
Histopathological slide image of lesion under (**A**) 0.4× and (**B**) 7× magnification of low-grade spindle cell, collagenous tumor.

Pathology for the 3.5 × 2.7 × 1.7 cm rubbery, pink-tan mass demonstrated a low-grade spindle cell, collagenous tumor (**Fig. 6A**, **6B**). Immunohistochemistry (IHC) showed spindle cells positive for CD34 and negative for S100, CD117, DOG1, desmin, smooth muscle actin, and HMB45. Less than 1% of the spindle cells were positive for Ki-67. No mitoses were identified. Solitary fibrous tumor was high in the differential based on pathological markers; however, IHC for STAT6 was negative. Therefore, the constellation of findings was most consistent with a NTF. Additionally, the patient tested negative for Gardner’s Syndrome.

Given the benign pathology, including an absence of high-grade features, location abutting but not invading the anal sphincter complex, and negative margins, no further treatment was required. The patient was scheduled for surveillance with pelvis MRI imaging every 6 months.

## DISCUSSION

Wire-needle localization with ultrasound guidance provides a new tool for the management of perirectal and perianal soft tissue masses. While this technique gained popularity in the field of breast surgery for the excision of deep, non-palpable breast lesions, our literature review revealed that the technique has been utilized in other fields, particularly thoracic surgery and head and neck surgery.^3–9)^ However, this is the first case reported to date highlighting its use and applicability to the field of colorectal surgery. Given the location of the mass and its close proximity to the anorectal sphincter complex, safe dissection to avoid iatrogenic injury was paramount in this case, particularly as it required dissection through a previously operated field. Performing dissection along the course outlined using an image-guided placed localizing technique allowed for not only safe, but also successful localization, and can serve as an option to future surgeons for similar lesions. While lesions in this region are rare, we hope highlighting this case provides a new tool for surgeons to utilize in similar cases in the future.

While this technique does offer the benefit of safe, guided dissection, it is not without risk. Injury to blood vessels, nerves, or the anorectal sphincter complex from the needle itself during placement of the wire is conceivable. Additionally, in this case there was a possibility that the lesion was malignant. Thus, there was the theoretical risk of tumor seeding as a result of puncturing the lesion with the wire during localization. While this is not the case in all malignant lesions, particularly in soft tissue masses that carry a risk of sarcoma, this risk must be considered. However, in this case, we felt that this was still the optimal approach in this patient, given the need to localize the lesion during dissection, which had previously proven to be extremely difficult.

Further exploring NTFs, they are benign fibrous tumors that predominantly occur along the posterior neck, up to one-third have been found in extra-nuchal locations.^13)^ They are more common in men, up to 80% of the time and are typically seen in those ranging from 10 to 60 years of age. They have been seen on the back along a para-spinal distribution, as well as the proximal extremities, along the shoulder, arms, thighs, and knees.^13)^ However, they have never been previously described occurring in the perirectal or perianal soft tissues. Given that nuchal and extra-nuchal lesions are histologically indistinguishable, they are both considered the same entity, “nuchal-type fibromas”. Regardless of location, these masses are typically palpable, firm but non-tender, slow-growing lesions, owing to their benign nature.^13)^

NTFs in any location are seen to be very rare. Michal et al. published the largest case series to date on 52 patients with NTFs. In the study, these tumors were evaluated histologically and found to typically comprise thick, interconnected collagen fibers to form a lobular-type architecture, interspersed with adipose tissue.^13–15)^ NFTs are best characterized by MRI. While some variability is seen, lesions on both T1 and T2-weighted images demonstrate low-signal intensity with mild, diffuse enhancement.^16)^ The lack of enhancement is attributed to their low cellularity with extensive fibrous collagen, and low water content.^14,17)^

While up to one-third of these tumors are found in extra-nuchal locations, very few have been reported in the pelvic region. One NTF was identified in the subcutaneous tissue of the right buttocks, with abutment but no invasion of the iliac bone or gluteus muscles.^2)^ Another NTF was diagnosed in the sacro-coccygeal region within the subcutaneous tissue adjacent to the coccyx, also without any bony or muscle invasion.^18)^ In a 51-year-old male with a history of Gardner’s Syndrome, a firm mass consistent with NTF was found on his left buttock extending to the gluteal cleft.^19)^

While these lesions appear to present sporadically, certain comorbidities have been observed more frequently in those with NTFs. Data from Michel et al. demonstrated that 44% of patients who developed NTFs had type II diabetes mellitus.^13)^ Numerous NTFs have also been reported in people who suffer repetitive trauma from activities such as weightlifting and contact sports.^16,20–22)^ Perhaps the most significant comorbidity associated with NTFs are in those with Gardner’s Syndrome (autosomal dominant Familial Adenomatous Polyposis associated with extracolonic tumors).^13,23–26)^ The patient did not have any preceding trauma or history of diabetes. Particularly, given the patient’s known recent history of 9 polyps being excised during colonoscopy, genetic testing for Gardner’s Syndrome was performed, and was negative.

## CONCLUSIONS

This case report describes the novel application of a hybrid surgical approach utilizing intraoperative image-guided localization for the successful localization and safe dissection with excision of a rare, non-palpable soft tissue mass in an atypical, difficult-to-access location. Procedural and technical considerations are outlined and described, as well as the pathology, NTF, which is uncommon to begin with and even more uncommon in extra-nuchal locations. We hope this publication can provide another tool in the arsenal of surgeons, whether it be in colorectal surgery or other operative fields, by highlighting the novel application of a multidisciplinary hybrid approach for soft tissue lesions in difficult-to-access areas.

## References

[1] Kapoor MM, Patel MM, Scoggins ME. The Wire and beyond: recent advances in breast imaging preoperative needle localization. Radiographics 2019; 39: 1886–906. 31560614 10.1148/rg.2019190041

[2] Davey MG, O’Donnell JPM, Boland MR, et al. Optimal localization strategies for non-palpable breast cancers -A network meta-analysis of randomized controlled trials. Breast 2022; 62: 103–13. 35151049 10.1016/j.breast.2022.02.004 PMC8844725

[3] Park JB, Lee SA, Lee WS, et al. Computed tomography-guided percutaneous hook wire localization of pulmonary nodular lesions before video-assisted thoracoscopic surgery: highlighting technical aspects. Ann Thorac Med 2019; 14: 205–12. 31333771 10.4103/atm.ATM_287_18 PMC6611205

[4] Wang Y, Jing L, Liang C, et al. Comparison of the safety and effectiveness of the four-hook needle and hook wire for the preoperative positioning of localization ground glass nodules. J Cardiothorac Surg 2024; 19: 35. 38297385 10.1186/s13019-024-02497-1 PMC10829251

[5] Sun X, Fu J, Ma C, et al. CT-guided microcoil versus hook-wire localization of pulmonary nodule prior to video-assisted thoracoscopic surgery without fluoroscopic guidance. BMC Pulm Med 2024; 24: 492. 39379924 10.1186/s12890-024-03306-0 PMC11463161

[6] Guo H, Ouyang Z, Li X, et al. Robotic-assisted CT-guided percutaneous pulmonary nodules localization by hook-wire needles: a retrospective observational study. J Thorac Dis 2024; 16: 4263–74. 39144352 10.21037/jtd-24-198 PMC11320270

[7] Winters R, Friedlander P, Noureldine S, et al. Preoperative parathyroid needle localization: a minimally invasive novel technique in reoperative settings. Minim Invasive Surg 2011; 2011: 487076. 22091358 10.1155/2011/487076 PMC3195344

[8] Thomas RH, Burke C, Howlett D. A technical note: pre-operative ultrasound-guided wire localization in head and neck surgery. Eur Arch Otorhinolaryngol 2011; 268: 743–6. 21400257 10.1007/s00405-011-1551-9

[9] Rozen WM, Cham A, Jones T. Managing suspicious cervical lymph nodes after thyroidectomy: the utility of hook-wire needle localization. ANZ J Surg 2010; 80: 299. 20575974 10.1111/j.1445-2197.2010.05259.x

[10] Finkelstein ER, Buitrago J, Jose J, et al. Lower extremity peripheral nerve pathology: utility of preoperative ultrasound-guided needle localization before operative intervention. Skeletal Radiol 2023; 52: 1997–2002. 37060462 10.1007/s00256-023-04347-y

[11] Thompson SM, Gorny KR, Jondal DE, et al. MRI-guided wire localization surgical biopsy in an adolescent patient with a difficult to diagnose case of lymphoma. Cardiovasc Intervent Radiol 2017; 40: 135–8. 27646518 10.1007/s00270-016-1464-5

[12] Mushtaq B, Myers R, Perrotti G, et al. Combined interventional radiology and surgical cut-down approaches for retained gallstones. Am Surg 2023; 89: 5002–4. 37283148 10.1177/00031348231180937

[13] Michal M, Fetsch JF, Hes O, et al. Nuchal-type fibroma: A clinicopathologic study of 52 cases. Cancer 1999; 85: 156–63. 9921988 10.1002/(sici)1097-0142(19990101)85:1<156::aid-cncr22>3.0.co;2-o

[14] Lee GK, Suh KJ, Lee SM, et al. Nuchal-type fibroma of the buttock: magnetic resonance imaging findings. Jpn J Radiol 2010; 28: 538–41. 20799020 10.1007/s11604-010-0459-4

[15] Balachandran K, Allen PW, MacCormac LB, Nuchal fibroma. A clinicopathological study of nine cases. Am J Surg Pathol 1995; 19: 313–317. 7872429

[16] Jayaram PR, Walsh J, Lari H, et al. Repetitive trauma-induced extra-nuchal-type fibroma. Skeletal Radiol 2022; 51: 681–5. 34554278 10.1007/s00256-021-03912-7 PMC8459702

[17] Samadi DS, McLaughlin RB, Loevner LA, et al. Nuchal fibroma: a clinicopathological Review. Ann Otol Rhinol Laryngol 2000; 109: 52–5. 10651413 10.1177/000348940010900110

[18] Shin JB, Son SW, Kim IH. Nuchal-type fibroma of the coccyx. Ann Dermatol 2008; 20: 41–4. 27303158 10.5021/ad.2008.20.1.41 PMC4904048

[19] Linos K, Sedivcova M, Cerna K, et al. Extra nuchal-type fibroma associated with elastosis, traumatic neuroma, a rare APC gene missense mutation, and a very rare MUTYH gene polymorphism: a case report and review of the literature. J Cutan Pathol 2011; 38: 911–8. 21752055 10.1111/j.1600-0560.2011.01745.x

[20] Sachs JP, Dardano AN. Nuchal-type fibroma induced by repetitive trauma from weightlifting: case report and comprehensive review of literature. Plast Reconstr Surg Glob Open 2024; 12: e5517. 38204868 10.1097/GOX.0000000000005517 PMC10781125

[21] Verdaguer-Faja J, Rodriguez-Garijo N, Arean-Cuns C, et al. Cutaneous ultrasound of the nuchal-type fibroma: diagnostic clues and surgery planning. J Ultrasound 2025; 28: 167–71. 38227145 10.1007/s40477-023-00842-z PMC11947352

[22] Bayne DR, Combes J, Pandya A. Nuchal fibrolipoma gives rugby players the ‘Hump’. ANZ J Surg 2016; 86: 416–8. 24888989 10.1111/ans.12697

[23] Michal M, Boudova L, Mukensnabl P. Gardner’s syndrome associated fibromas. Pathol Int 2004; 54: 523–6. 15189507 10.1111/j.1440-1827.2004.01660.x

[24] Diwan AH, Graves ED, King JA, et al. Nuchal-type fibroma in two related patients with Gardner’s syndrome. Am J Surg Pathol 2000; 24: 1563–7. 11075861 10.1097/00000478-200011000-00015

[25] Dawes LC, La Hei ER, Tobias V, et al. Nuchal fibroma should be recognized as a new extracolonic manifestation of Gardner-varient familial adenomatous polyposis. Aust N Z J Surg 2000; 70: 824–6. 11147449 10.1046/j.1440-1622.2000.01958.x

[26] Kiessling P, Dowling E, Huang Y, et al. Identification of aggressive Gardner syndrome phenotype associated with a de novo APC variant, c.4666dup. Cold Spring Harb Mol Case Stud 2019; 5: a003640. 30696621 10.1101/mcs.a003640 PMC6549566

